# Metabolic flux reprogramming in *Mycobacterium tuberculosis*-infected human macrophages

**DOI:** 10.3389/fmicb.2023.1289987

**Published:** 2023-11-17

**Authors:** Khushboo Borah Slater, Luana Moraes, Ye Xu, Daniel Kim

**Affiliations:** ^1^School of Biosciences, University of Surrey, Guildford, United Kingdom; ^2^Laboratório de Desenvolvimento de Vacinas, Instituto Butantan, São Paulo, Brazil; ^3^Programa de Pós-Graduação Interunidades em Biotecnologia-USP, São Paulo, Brazil

**Keywords:** tuberculosis, immunometabolism, *Mycobacterium tuberculosis*, fluxomics, human macrophages

## Abstract

Metabolic fluxes are at the heart of metabolism and growth in any living system. During tuberculosis (TB) infection, the pathogenic *Mycobacterium tuberculosis* (Mtb) adapts its nutritional behaviour and metabolic fluxes to survive in human macrophages and cause infection. The infected host cells also undergo metabolic changes. However, our knowledge of the infected host metabolism and identification of the reprogrammed metabolic flux nodes remains limited. In this study, we applied systems-based ^13^C-metabolic flux analysis (MFA) to measure intracellular carbon metabolic fluxes in Mtb-infected human THP-1 macrophages. We provide a flux map for infected macrophages that quantified significantly increased fluxes through glycolytic fluxes towards pyruvate synthesis and reduced pentose phosphate pathway fluxes when compared to uninfected macrophages. The tri carboxylic acid (TCA) cycle fluxes were relatively low, and amino acid fluxes were reprogrammed upon Mtb infection. The knowledge of host metabolic flux profiles derived from our work expands on how the host cell adapts its carbon metabolism in response to Mtb infection and highlights important nodes that may provide targets for developing new therapeutics to improve TB treatment.

## Introduction

Tuberculosis (TB) remains a significant global health concern. COVID-19 pandemic negatively impacted TB diagnosis, treatment, and control (Pai et al., 2022). TB continues to spread, resulting in 10.6 million new cases in 2021 and 1.6 million deaths, reversing the years of slow decline in TB cases. The emergence of drug-resistant TB and the ineffectiveness of the current treatments pose a serious public health threat (World Health Organization, 2022). It is therefore urgent to identify new targets for drug development.

The causative agent of TB, *Mycobacterium tuberculosis* (Mtb) is a highly adaptable pathogen. Macrophages, the phagocytic innate immune cells, are one of the primary residing sites for Mtb (Howard and Khader, 2020). The interactions between host macrophages and Mtb are a primary determinant of the outcome of infection (Kumar et al., 2019; Howard and Khader, 2020). We know that the pathogenic Mtb acquires multiple nutrient sources including amino acids and cholesterol from the host macrophages for intracellular replication (Pandey and Sassetti, 2008; Beste et al., 2013; Borah et al., 2019a,b). Thus, Mtb relies on host cell metabolites for its growth and survival during infection. Whilst Mtb’s *in vitro* and *in vivo* metabolism has been extensively investigated, our understanding of the infected host cell metabolism remains underexplored.

Macrophage polarisation into the M1 pro-inflammatory or M2 anti-inflammatory phenotype is tightly linked to cellular metabolism (Howard and Khader, 2020). M1 macrophages use glycolysis for energy production, whiilst M2 macrophages rely on mitochondrial oxidative phosphorylation and fatty acid oxidation (Howard and Khader, 2020). Metabolic reprogramming in macrophages is important to regulate immune functions such as the production of cytokines and antimicrobial responses to eliminate Mtb infection (Kumar et al., 2019). Upon Mtb infection, macrophages acquire a M1-like phenotype with increased glycolysis and increased production of proinflammatory cytokines (Shi et al., 2016; Kumar et al., 2019; Howard and Khader, 2020). Previous studies have demonstrated that Mtb infection led to a metabolic switch to aerobic glycolysis in infected macrophages, like the Warburg effect described in cancer (Gleeson et al., 2016; Sheedy and Divangahi, 2021). The increase in glycolysis was accompanied by a downregulation of the tricarboxylic acid (TCA) cycle, and oxidative phosphorylation (OXPHOS) (Shi et al., 2016). Hypoxia-inducible factor 1 (HIF-1α) has gained significant interest due to its role in macrophage polarisation and regulation of glucose metabolism (Wang et al., 2017; Terán et al., 2022). Lactate production through the oxidation of glycolytically-derived pyruvate was significantly elevated in Mtb infected macrophages and mice (Shin et al., 2011; Shi et al., 2016). Mtb infection also induced host cell mitochondrial metabolic reprogramming. Mtb infection downregulated mitochondrial bioenergetics in THP-1 and human monocyte-derived macrophages (Cumming et al., 2018). An Mtb effector protein encoded by Rv1813c altered host mitochondrial function by inhibiting cytochrome c release from mitochondria and causing delayed apoptosis (Martin et al., 2023). Glutamine and arginine amino acid metabolism are important in regulating macrophage polarisation (Kim et al., 2020; Jiang et al., 2022). Immunometabolism is therefore an important research area that has received growing interest over the recent years to understand infected host metabolic reprogramming and develop innovative host-directed therapeutics (HDTs). To develop effective HDTs, it is crucial to know precisely which metabolic nodes are reprogrammed in infected macrophages. For example, statins are proposed adjuvants, but their molecular basis for anti-TB activity needs to be established (Dutta et al., 2020). Host cell glycolysis is an attractive target (Kim et al., 2020). However, glycolysis is central for cellular metabolism and for fuelling anti-TB responses, so inhibition or elevation of all or specific glycolytic nodes or enzymes will need to be carefully considered. Although some progress has been made to identify overall changes in the metabolic pathways of infected cells, there is still no information on the intracellular fluxes that drive pathway activity.

Metabolic fluxes are at the heart of driving cellular metabolism and metabolic reprogramming (Borah et al., 2021b). The intracellular carbon fluxes, core to sustaining cellular functions have not yet been measured. In this study, we applied fluxomics using ^13^C-Metabolic Flux Analysis (MFA) to measure intracellular carbon metabolic fluxes of Mtb infected human macrophages. Our aim was to establish which fluxes or nodes in central carbon metabolism involving glycolysis, pentose phosphate pathway (PPP) and tricarboxylic acid (TCA) cycle were significantly reprogrammed upon Mtb infection. Here we provide the first measurements of carbon metabolic fluxes of Mtb-infected macrophages and highlight significantly reprogrammed fluxes that could be further investigated for potential HDT development.

## Materials and methods

### Model organisms

*Mycobacterium tuberculosis* (Mtb) H37Rv was the mycobacterial strain used in the study (Borah et al., 2019a,b). The THP-1 monocytic cell line was used as the human macrophage model used in this work (Borah et al., 2019a,b).

### *Mycobacterium tuberculosis* H37Rv growth

Mtb H37Rv was cultured using Middlebrook 7H11 agar (Merck) and Middlebrook 7H9 broth (Merck) containing 10% (vol/vol) oleic acid-albumin-dextrose-catalase enrichment medium supplement (OADC) (Becton Dickenson) and 0.5% (vol/vol) glycerol (Merck). Mtb was streaked onto brain heart infusion agar (Merck) plates to check the purity of cultures prior to infection assays.

### THP-1 cell culture

THP-1 monocytic cell line was grown in RPMI 1640 growth media (Merck) supplemented with 10% heat inactivated fetal bovine serum (FBS) (Merck) at 37°C, 5% CO_2_ and 95% humidity. For ^13^C labelling experiments, modified RPMI growth media (Gibco™ 11,879,020) without glucose was prepared by adding 100% [U-^13^C_6_]glucose (w/v, CK Isotopes).

### [U-^13^C_6_]glucose isotopic labelling assays

Isotopic labelling experiments were conducted by growing THP-1 macrophages in RPMI media containing 100% [U-^13^C_6_]glucose. Briefly, monocytes were grown in unlabelled RPMI media followed by differentiation into macrophages for three days using 50 nM Phorbol 12-myristate 13-acetate (PMA) in 175 cm^2^ tissue culture flasks and in 6 well plates. Around 3 × 10^7^ and 1 × 10^6^ macrophages were cultivated in 175cm^2^ flasks and 6 well plates, respectively.

### Infection of THP-1 macrophages with Mtb H37Rv

THP-1 monocytes were differentiated into macrophages using PMA in RPMI media containing 100% [U-^13^C_6_]glucose as described in the previous section. On the day of infection, macrophages were cultivated either in 175 cm^2^ tissue culture flasks or 6 well plates and washed with phosphate buffered saline (PBS) supplemented with 0.49 mM Mg2+ and 0.68 mM Ca2+ (PBS^+^) three times prior to infection (Borah et al., 2019a). Mtb broth cultures were grown in 7H9 media with 10% OADC and 0.5% glycerol. Exponentially growing Mtb cultures were washed three times with PBS and added to macrophages at a multiplicity of infection (MOI) of 5. Macrophages infected with Mtb were incubated at 37°C, 5% CO_2_ and 95% humidity for 3–4 h, followed by washing the macrophages with PBS^+^ three times. Fresh media with 100% [U-^13^C_6_]glucose was added to infected macrophages and left for 48 h prior to cell harvest for metabolomics and isotopomer analysis. Uninfected macrophages were washed and incubated for 48 h in 100% [U-^13^C_6_]glucose containing RPMI as controls.

### Metabolomics and ^13^C isotopic labelling analysis

After 48 h of infection, uninfected and infected macrophages cultivated in 6-well plates were washed twice with PBS and cells were scraped. Cells were spun at 300 g for five minutes. 6 M hydrochloric acid (HCl) was added to the cells and boiled for 10 min (Borah et al., 2019a). Amino acid hydrolysates were prepared from cell lysates by incubating the lysates in 6 M HCl at 100°C for 24 h. Gas-chromatography mass spectrometry (GC–MS) metabolomics was performed to measure amino acid pool sizes and for mass isotopomer analysis. Amino acid hydrolysates were dried and derivatized using pyridine and tert-butyldimethyl silyl chloride (TBDMSCl) (Merck) (Borah et al., 2019a). Amino acids were analyzed using a VF-5 ms inert 5% phenyl-methyl column (Agilent Technologies) on GC–MS. Mass spectra were extracted using chemstation GC–MS software. Mass spectrometry datasets were corrected for natural isotope effects using the Isotopomer network compartmental analysis (INCA) platform (Young, 2014; Borah et al., 2021a). Average ^13^C in an amino acid was calculated from the fractional abundance of the mass isotopomers in the amino acid fragments. The corrected mass isotopomer distributions (MIDs) for non-essential amino acids and organic acids were included into the macrophage metabolic model for flux estimation using ^13^C-MFA.

### Biomass measurements

Biomass measurements were calculated for uninfected and infected macrophages. 3 × 10^7^ macrophages cultivated in 175 cm2 tissue culture flasks were used for biomass assays. For calculating dry cell weight, macrophage samples were distributed into eppendorfs, freeze dried and weighed. Dry cell pellets were used to measure protein using BCA (bicinchoninic acid) protein determination kit (Merck), lipids using lipid Assay Kit (Abcam ab242305), total DNA using Qubit dsDNA BR Assay (Fisher 10,146,592), total RNA using QUANT-IT RNA BR assay kit (Fisher 10,266,793) and carbohydrates using carbohydrate kit (Merck, MAK104). Amino acids were quantified using GC–MS method as described in the previous section.

### ^13^C-MFA

Metabolic flux estimations were performed using INCA, version 2.2 (Young, 2014). The MIDs included in the model for flux estimation are provided in Supplementary File S2. Uninfected and infected macrophages were at pseudo-steady state (metabolic and isotopic) after 48 h of ^13^C-labelling and Mtb-infection (Beste et al., 2013; Borah et al., 2019a). Fluxes were estimated using a non-linear weighted least squares fitting approach to calculate net and exchange flux distributions that were the best-fit for generation of the isotopic labelling data and biomass constraints (Young, 2014; Borah et al., 2021a). A random initial guess and multistart optimisation approach with 200 restarts was used for flux estimations. Flux profiles with the minimum statistically acceptable SSR were considered the best-fit. The goodness of fit of the flux maps was assessed by comparing the simulated and experimental measurements. The uncertainties in the flux estimations were checked using the Monte Carlo analysis function built into INCA. The confidence intervals and frequency distribution plots were calculated using Monte Carlo analysis, with significance level α = 0.05, 100 trials per iteration and a relative error tolerance of 0.005.

### Statistical analysis

Statistical analysis including chi-square test and Monte Carlo analysis was performed using INCA and t-tests were calculated using Graphpad version 8.2.1.

### Metabolic modelling

**Figure 1:**
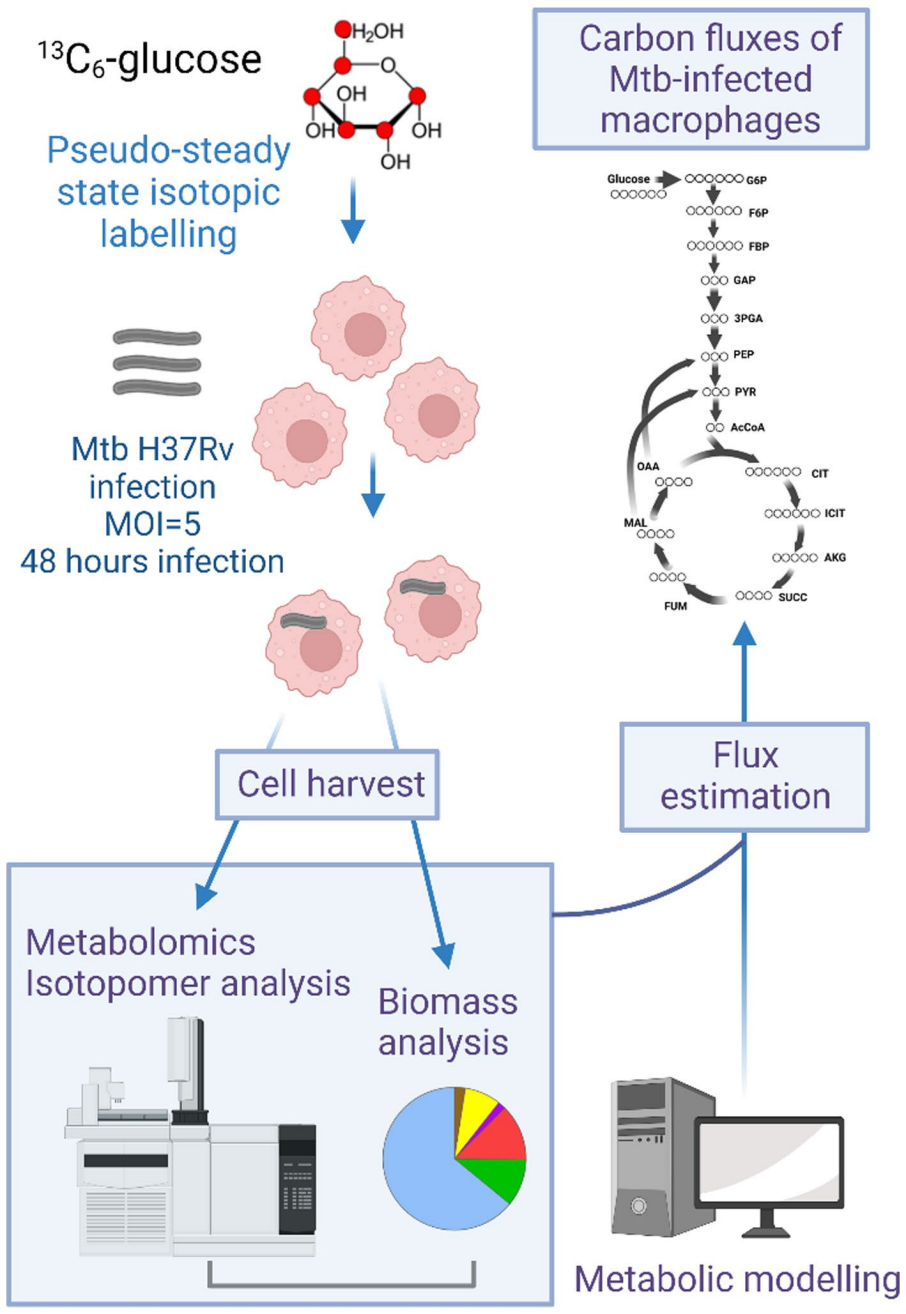
Overview of the method for conducting ^13^C-metabolic flux analysis (MFA) in human THP-1 macrophages. Macrophages were grown in [U-^13^C_6_]glucose containing RPMI media for 72 h followed by infection with Mtb H37Rv for 48 h. After infection macrophages were harvested for metabolomics and isotopomer analysis using gas chromatography mass spectrometry (GC–MS). An isotopomer network model of human macrophages containing central carbon metabolic reactions was constructed. Metabolomics and isotopomer data were incorporated into the model and flux estimation was done using INCA.

Metabolic modelling of a macrophage was conducted using INCA, version 2.2 (Young, 2014). The isotopomer model consists of a central carbon metabolic network with 57 net flux reactions and 25 exchange fluxes (Supplementary File S1). Metabolic reactions include glycolysis, gluconeogenesis, the PPP, the TCA cycle, anaplerosis, biomass reaction and amino acid synthesis (glutamate, glutamine, proline, arginine, aspartate, asparagine, alanine, serine, glycine, cysteine, and tyrosine). The biomass reaction used to constrain the model was constructed from the experimentally measured biomass proportions of uninfected and infected macrophages (Figure 1). The network also consists of uptake reactions for glucose and amino acids: glutamine, alanine, glycine, serine, tyrosine, aspartate, asparagine, cysteine, arginine, glutamate, and proline. The glucose uptake flux was fixed at 100 and fluxes were estimated relative to the glucose uptake rate.

## Results

### ^13^C-MFA workflow for human macrophages

This section outlines the methodology for conducting ^13^C-MFA in THP-1 macrophages (uninfected or infected with Mtb H37Rv) (Figure 1). [U-^13^C_6_]glucose was chosen as the isotopic substrate for the labelling experiment. THP-1 monocytes were differentiated into macrophages with PMA in RPMI growth media containing 100% [U-^13^C_6_]glucose for 72 h. We did not find any significant increase in ^13^C incorporation into the biomass of macrophages when monocytes were cultivated in [U-^13^C_6_]glucose for 48 h prior to differentiation into macrophages. Hence, we chose to grow macrophages in ^13^C labelling media during differentiation and infection. Macrophages were uninfected (control) or infected with Mtb H37Rv for 48 h in ^13^C labelling media. The infection period of 48 h was chosen as the pseudo steady-state labelling period in infected macrophages, as established from our previously published work (Beste et al., 2013; Borah et al., 2019a). Uninfected or infected macrophages were harvested after 48 h for metabolomics and isotopic labelling analysis. In parallel, biomass composition analyses were conducted for uninfected and Mtb-infected macrophages grown in unlabelled RPMI media. The biomass composition was used to construct the biomass equation that constrained the isotopomer metabolic network model which was constructed to include the central carbon metabolic reactions for glycolysis/ gluconeogenesis, pentose phosphate pathway (PPP) and tricarboxylic acid (TCA) cycle. The model was constructed based on published literature and databases including HumanCyc and KEGG (Amaral et al., 2011; Duckwall et al., 2013; Antoniewicz, 2018; Lagziel et al., 2019). The isotopomer data from the labelling experiments were incorporated into the model and computationally fitted using the MATLAB-based isotopomer network compartmental analysis (INCA) platform to compute the best-fit flux maps.

### Mtb infection induces changes in biomass production by macrophages

**Figure 2:**
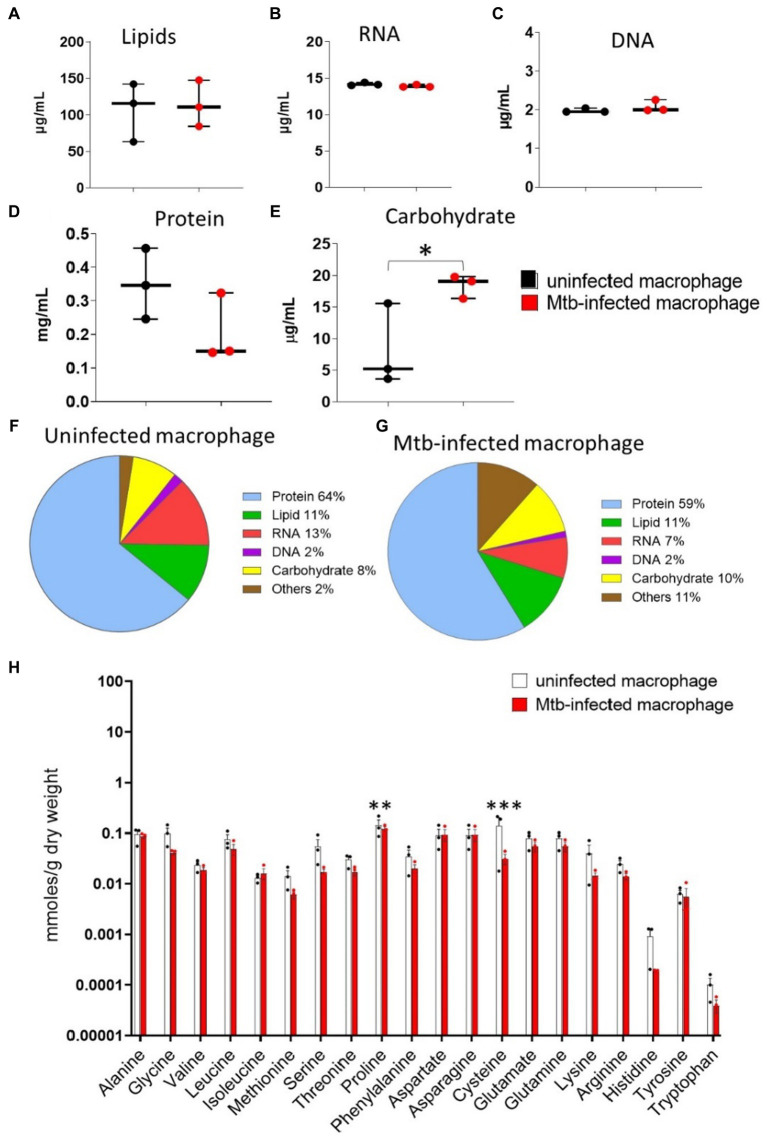
Macromolecular biomass composition measurements. Uninfected and Mtb-infected macrophages (post 48 h of infection) were harvested for measuring cell dry weight, **(A)** lipids, **(B)** RNA, **(C)** DNA, **(D)** protein and **(E)** carbohydrates. **(F,G)** Show the proportional biomass composition in uninfected and infected macrophages. Values are mean ± standard error of the mean (SEM) from three independent infections. Statistical significance is determined without correction for multiple comparisons, with alpha = 0.05; * indicates statistical significance, *, *p* < 0.05. **(H)** Cellular amino acid pool sizes for uninfected and Mtb-infected macrophages. Quantification was determined using GC–MS metabolomics. Values are mean ± standard error of the mean (SEM) from three to four independent infections. Statistical significance determined without correction for multiple comparisons, with alpha = 0.05; * indicates statistical significance; **, *p* < 0.005; ***, *p* < 0.0005.

Biomass measurements were used to constrain the metabolic model for flux estimations. We measured macromolecular biomass composition: nucleic acids, protein, lipids, and carbohydrates of uninfected and Mtb-infected macrophages (Figures 2A–E). Protein accounted for the highest proportion of cell dry weight (>50%), followed by lipids and carbohydrates (Figures 2F,G). There were no significant changes in total cellular lipids, protein, RNA and DNA upon Mtb infection. Total cellular carbohydrates in infected macrophages were significantly higher when compared to uninfected macrophages demonstrating the shift in carbohydrate synthesis upon Mtb infection (Figure 2E).

We quantified cellular amino acid pools using GC–MS metabolomics to identify changes induced in infected macrophages (Figure 2H). There were no significant differences in the pool sizes of essential amino acids (valine, leucine, isoleucine, methionine, threonine, lysine, histidine, tryptophan, phenylalanine) between the two groups as these are not synthesised in human cells but taken up from the growth media. Amongst the non-essential amino acids, proline and cysteine levels were significantly lower in infected macrophages. Cysteine is synthesised directly from serine; the pool size of cellular serine was over two-fold lower in infected macrophages. Aspartate and glutamate, the two primary precursors for amino acid biosynthesis did not change significantly upon Mtb-infection.

### Mtb-infected macrophages show distinct ^13^C isotopomer profiles of amino acids

**Figure 3:**
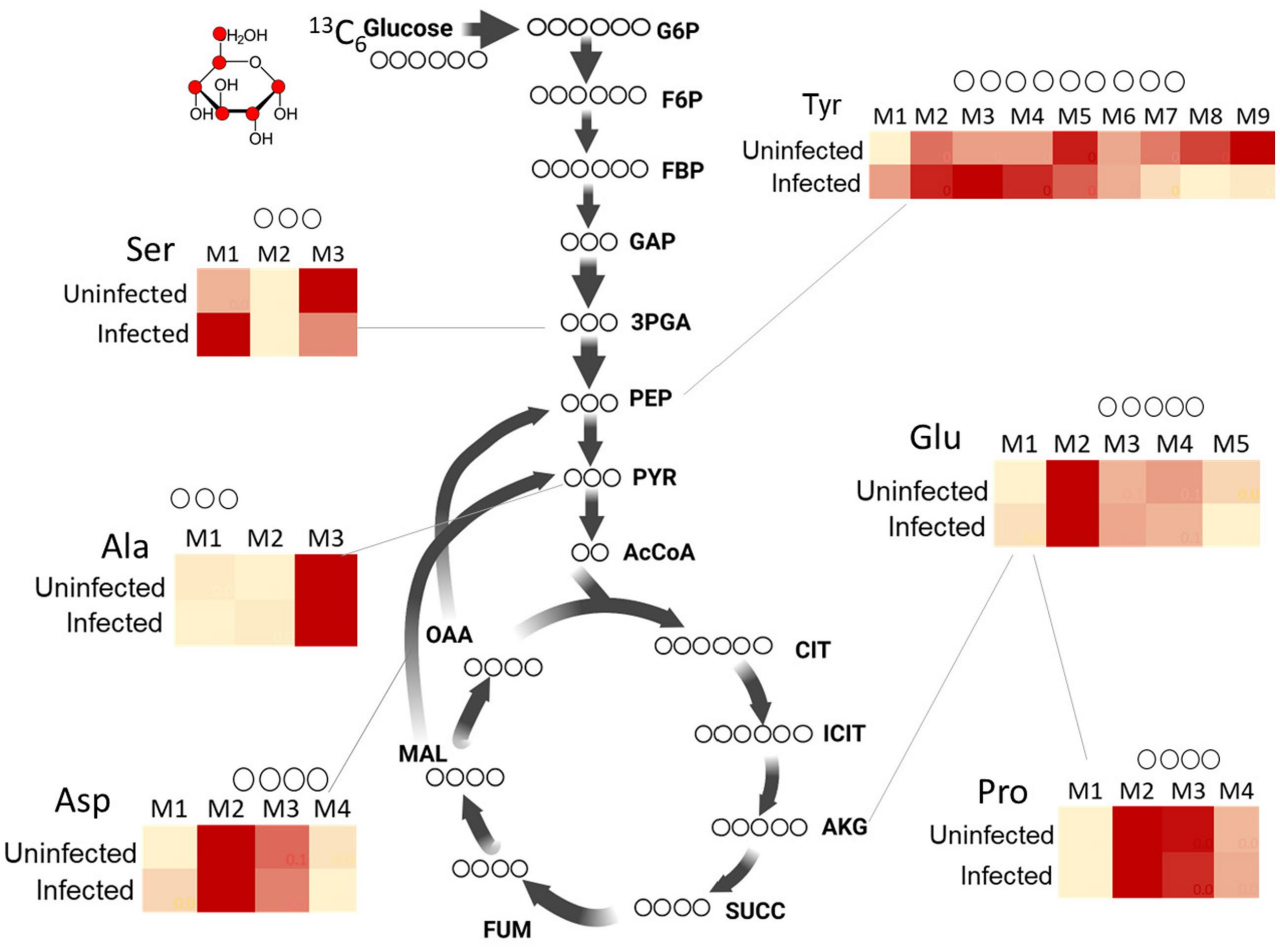
^13^C isotopomer profiles of amino acids from uninfected and Mtb-infected macrophages. Comparison of ^13^C distribution between uninfected vs. infected macrophages are shown for Ser (serine), Tyr (tyrosine), Ala (alanine), Asp (aspartate), Glu (glutamate) and Pro (proline). M1, M2, M3….M9 are carbon mass isotopomers. Values are mean ± standard error of the mean (SEM) from three independent infections.

**Figure 4:**
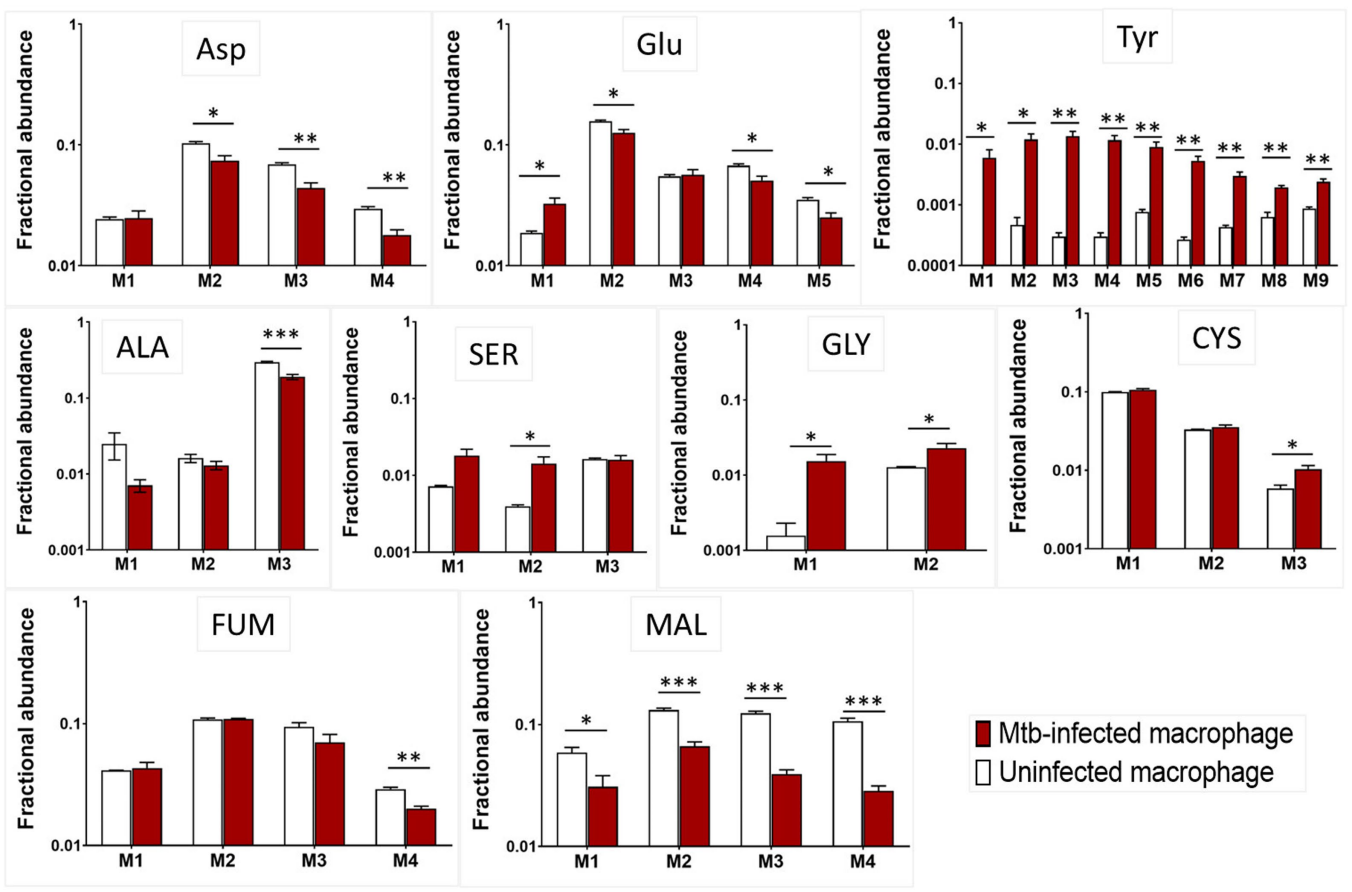
^13^C incorporation in amino acids from uninfected and Mtb-infected macrophages. M1, M2, M3….M9 are carbon mass isotopomers shown for alanine (Ala), glutamate (Glu), tyrosine (Tyr), serine (Ser), glycine (Gly), cysteine (Cys), aspartate (Asp), fumarate (Fum) and malate (Mal). Values are mean ± standard error of the mean (SEM) from three independent infections.

**Figure 5:**
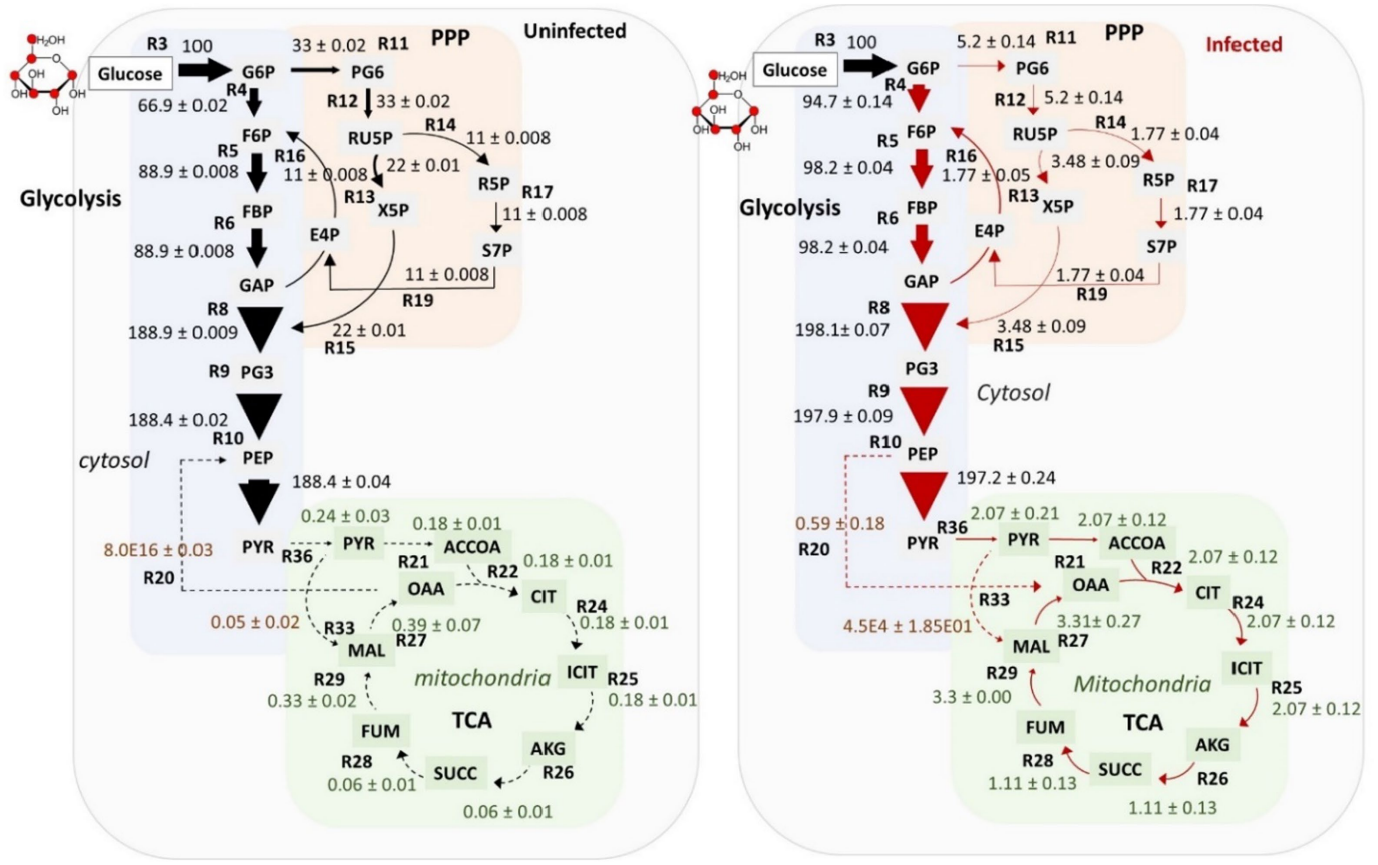
Flux maps of uninfected and infected macrophages. Fluxes were calculated relative to the glucose uptake rate set to 100. Fluxes are shown by arrows for the central carbon metabolic pathways: glucose/gluconeogenesis, pentose phosphate pathway (PPP), tri carboxylic acid (TCA) cycle and anaplerotic reactions. Fluxes are proportional to the thickness of the arrow. G6P (glucose 6-phosphate), F6P (fructose 6-phosphate), FBP (fructose 1,6-bisphosphate), GAP (glyceraldehyde 3-phosphate), PG3 (3-phophoglycerate), PEP (phosphoenolpyruvate), PYR (pyruvate), MAL (malate), OAA (oxaloacetate), SUCC (succinate), ACCOA (acetyl coenzyme A), ICIT (isocitrate), AKG (α-ketoglutarate), FUM (fumarate), PG6 (6-phosphogluconic acid), X5P (xylulose 5-phosphate), E4P (erythrose 4-phosphate), R5P (ribose 5-phosphate), RU5P (ribulose 5-phosphate) and S7P (sedoheptulose 7-phosphate). Fluxes are marked as R3, R4, R5….R36. See Supplementary File S3 for the list of all fluxes and their best-fit estimated values.

In this section, we present the ^13^C mass isotopomer distribution profiles for macrophage amino acids. The carbon backbone in amino acids is synthesised from metabolic intermediates generated from central carbon metabolism. The carbon backbone of alanine (Ala) and serine (Ser) are produced from glycolytic intermediates pyruvate and 3-phosphoglyceric acid, respectively. The ^13^C distribution profile for Ser is strikingly different for infected macrophages; the proportional distribution of ^13^C is highest in isotopomer family M3 for uninfected macrophages but M1 for infected macrophages (Figure 3). Quantitative analysis in Figure 4 shows that the fractional abundance or proportion of ^13^C in M2 were significantly higher for infected macrophages. Glycine (Gly) and cysteine (Cys) which were synthesised from Ser also showed higher proportions of M2 and M3 in infected macrophages. This was expected as these three amino acids are synthesised from the same carbon precursor derived through glycolysis. The proportion of ^13^C in Ala M3 is higher in uninfected macrophages (Figure 4). The differences in these amino acids indicate likely changes in carbon fluxes that generated respective metabolic precursors through glycolysis in infected macrophages. Tyrosine is derived from the PPP and glycolytic intermediates; the ^13^C distribution profile is significantly different between uninfected and infected groups (Figures 3, 4) indicating changes in fluxes through these central pathways. The TCA cycle derived intermediates fumarate (FUM) and malate (MAL) and amino acids aspartate (Asp) and glutamate (Glu) exhibited quantitatively distinct proportional ^13^C in infected macrophages (Figure 5). Although the total cellular pool sizes of Asp., Glu and Tyr showed no significant differences between uninfected and infected macrophages, the ^13^C isotopomer analysis was distinct between the two groups; this confirms that the measurement of pool sizes alone may not be an accurate indicator of changes in carbon fluxes through a pathway and ^13^C isotopomer analyses could provide more detailed information. The mass isotopomer distribution profiles obtained from the labelling experiments were incorporated into the metabolic model to estimate carbon fluxes through the central metabolic network, which are presented in the next section.

### Carbon metabolic flux phenotype of uninfected and Mtb-infected macrophages

Metabolic fluxes for central carbon metabolism (CCM) were quantified using ^13^C-MFA (see Figure 1 for the overview of the steps for MFA). The details of reactions included in the isotopomer network model of a macrophage are provided in Supplementary File S1. Fluxes were resolved for the pathways involved in CCM: glycolysis/gluconeogenesis, PPP, and the TCA cycle (Supplementary File S3). The flux maps for uninfected and infected macrophages are shown in Figure 5. Fluxes were quantified relative to the glucose uptake rate set arbitrarily at 100. The uptake of glucose in macrophages has been reported to be mediated by GLUT1, a member of the SLC2 transporter family (Nonnenmacher and Hiller, 2018). In the cytosol, glucose is converted to glucose 6 phosphate (G6P) by hexokinase (Nonnenmacher and Hiller, 2018). Flux from G6P was partitioned to glycolysis via fructose 6 phosphate (F6P) and to PPP via 6-phosphogluconic acid (PG6). Reactions R4 to R10 of glycolysis carried the highest carbon fluxes relative to the PPP and the TCA cycle. The uninfected and infected macrophages profile exhibited oxidative TCA cycle fluxes. The TCA cycle fluxes were relatively lower than that in glycolysis and the PPP. Anaplerotic carbon metabolism was modelled between phosphoenolpyruvate (PEP) and oxaloacetate (OAA) and between pyruvate (PYR) and malate (MAL). The cytosolic and mitochondrial pools of OAA and MAL were lumped together to improve flux resolution in these reactions. Anaplerotic reactions carried the lowest carbon flux relative to the rest of the central pathways. We compared the best-fit carbon flux estimates between uninfected and infected macrophages and described the significantly altered pathways in the next section.

### Glycolytic and PPP carbon fluxes were altered in Mtb-infected macrophages

**Figure 6:**
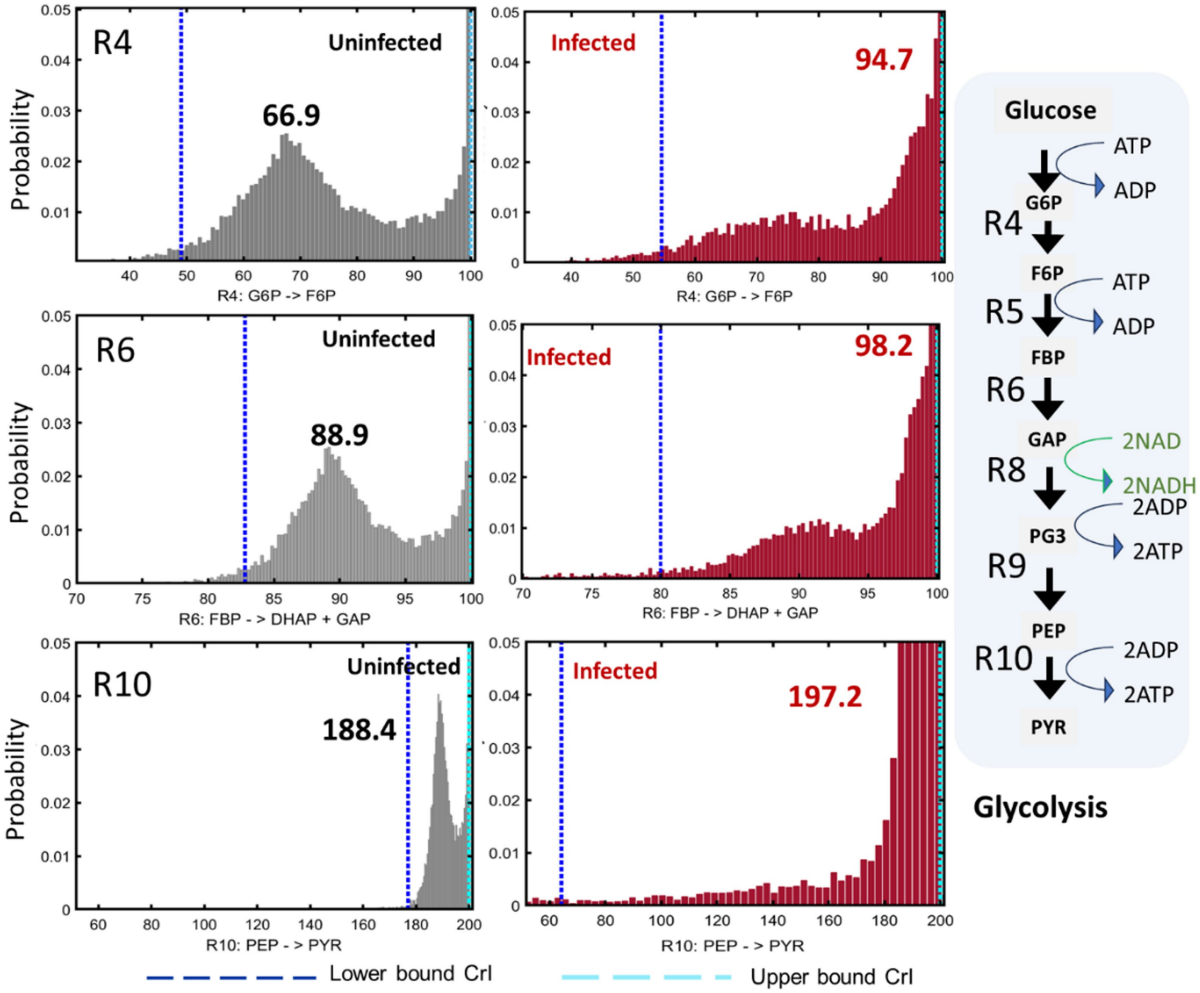
Glycolytic fluxes in uninfected and infected macrophages. The frequency distribution plots and upper and lower confidence intervals (CrI) for reactions R4 (G6P - > F6P), R6 (FBP - > DHAP + GAP) and R10 (PEP - > PYR) are shown. R4, R5.…R10 are reactions in glycolysis. ATP, ADP, NAD and NADH are indicated next to the reactions that either consume or generate these coenzymes and cofactors. G6P (glucose 6-phosphate), F6P (fructose 6-phosphate), FBP (fructose 1,6-bisphosphate), DHAP (dihydroxyacetone phosphate), GAP (glyceraldehyde 3-phosphate), PEP (phosphoenolpyruvate) and PYR (pyruvate). ^13^C isotopomer data of uninfected and infected macrophages (*n* = 3) were used for ^13^C-MFA. Best-fit flux estimates were calculated relative to the glucose uptake rate (set at 100) using MATLAB-based isotopomer network compartmental analysis (INCA) platform. Monte Carlo simulations were performed using INCA to compute the frequency distribution plots and lower and upper confidence levels (Crl) for the best-fit fluxes.

The statistical significance of the differences between the fluxes of Mtb-infected and uninfected macrophages was assessed using Monte Carlo analysis. The frequency distribution plots for glycolytic fluxes are shown in Figure 6 and Supplementary Figure S1. Glycolytic fluxes R4, R5, R6, R7, R8, R9 and R10 were significantly higher in infected macrophages. Glycolysis is a faster route for ATP production and generation of biosynthetic precursors, which are required to mount host defence responses against Mtb (Shi et al., 2016). We have selectively illustrated the statistical comparisons for fluxes R4, R6 and R10 in Figure 6. Flux R4 from G6P to F6P, the first step of glycolysis was significantly higher in infected macrophages. The plots for R4 are significantly different between uninfected and infected macrophages. For uninfected macrophages, the maximum probable and best-fit net flux value of R4 is 66.9, but for infected macrophages, this value is 94.7. Similarly, the plots for R6 (from fructose 1,6-bisphosphate (FBP) to glyceraldehyde 3-phosphate (GAP) and dihydroxyacetone phosphate (DHAP)) show the maximum probable best-fit flux of 98.2 for R6 in infected macrophages vs. 88.9 in uninfected macrophages. The comparisons for rest of the glycolytic fluxes R5, R7, R8 and R9 are shown in Supplementary Figure S1 which also shows significant changes in uninfected vs. infected macrophages. The plots for R10, the last step rate-limiting step of glycolysis from PEP to PYR catalysed by the pyruvate kinase enzyme show a maximum flux of 197.2 for infected macrophages vs. 188.4 in uninfected macrophages. Carbon flux from PYR is withdrawn for the synthesis of amino acids including ALA and lactate. The statistical resolution for these output fluxes was not refined, however the total flux withdrawn from PYR to synthesise ALA and lactate was higher in infected macrophages (195.19 in infected vs. 188.20 in uninfected macrophages).

**Figure 7:**
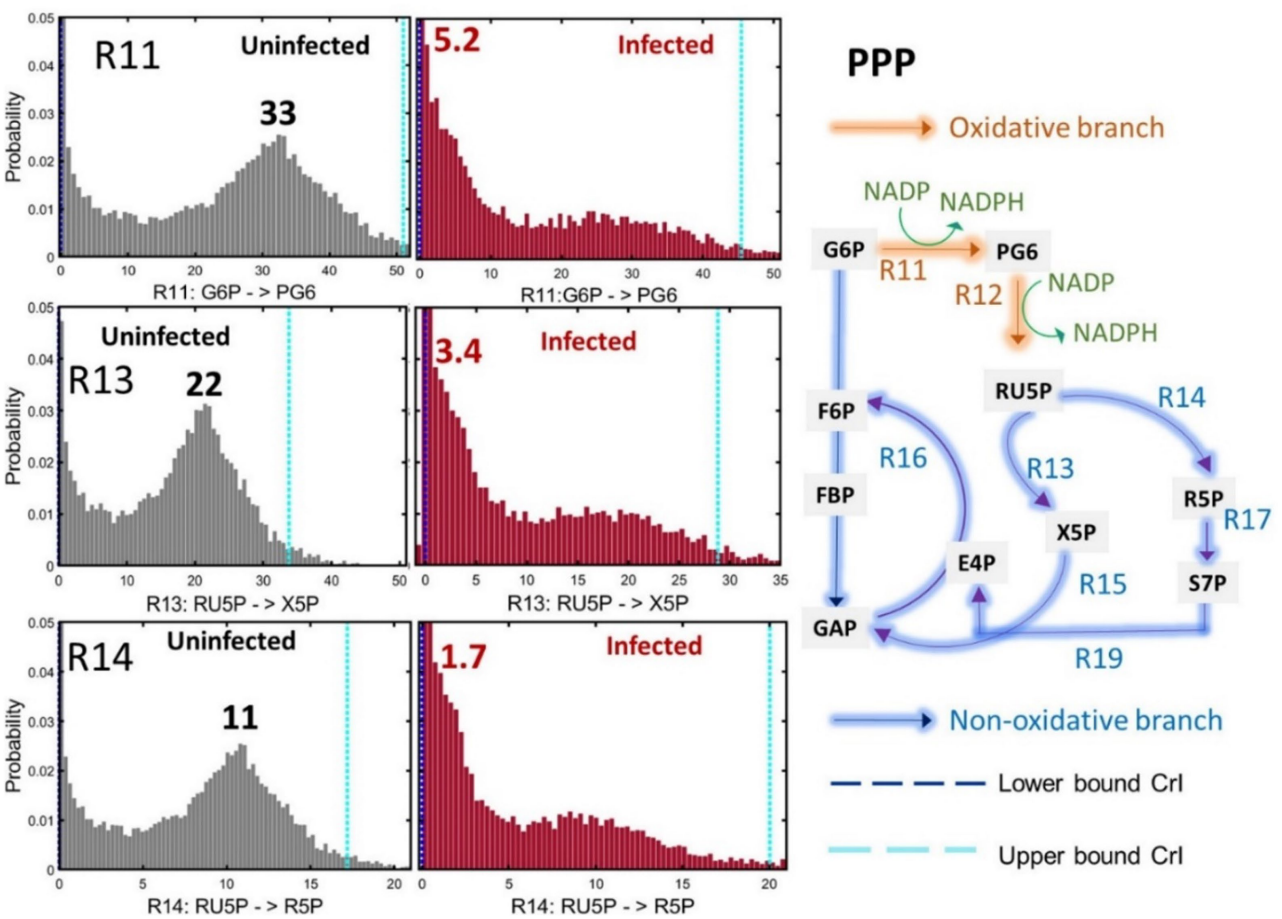
Pentose phosphate pathway (PPP) fluxes in uninfected and infected macrophages. The frequency distribution plots and upper and lower confidence intervals (CrI) for reactions R11 (G6P - > PG6), R13 (RU5P - > X5P) and R14 (RU5P - > R5P) are shown. Oxidative and non-oxidative reactions of the PPP (R11…R19) are shown in the pathway outline. NADP and NADPH indicated next to the reactions R11 and R12 are generated by oxidative branch of the PPP. G6P (glucose 6-phosphate), F6P (fructose 6-phosphate), FBP (fructose 1,6-bisphosphate), GAP (glyceraldehyde 3-phosphate), PG6 (6-phosphogluconic acid), X5P (xylulose 5-phosphate), E4P (erythrose-4-phosphate), R5P (ribose 5-phosphate), RU5P (ribulose 5-phosphate) and S7P (sedoheptulose-7-phosphate). 13C isotopomer data of uninfected and infected macrophages (*n* = 3) were used for 13C-MFA. Best-fit flux estimates were calculated relative to the glucose uptake rate (set at 100) using MATLAB-based isotopomer network compartmental analysis (INCA) platform. Monte Carlo simulations were performed using INCA to compute the frequency distribution plots and lower and upper confidence levels (Crl) for the best-fit fluxes.

The fluxes for both oxidative and non-oxidative branches of the PPP were significantly reduced in Mtb-infected macrophages. The plots for flux R11 (the first step of the PPP: G6P to PG6) and R12 (PG6 to ribulose 5-phosphate (RU5P)) show the best-fit flux value of 33 in uninfected macrophages vs. 5.2 in infected macrophages (Figure 7; Supplementary Figure S2). These oxidative PPP fluxes generate NADPH coenzymes that fuel cellular lipid synthesis. The fluxes through the non-oxidative branches of the PPP (R13, R14, R15, R16, R17, R18, R19) were also reduced in infected macrophages (Figure 7; Supplementary Figure S2). The plots for R13 and R14 in Figure 7 shows over a five-fold reduction of fluxes in infected macrophages and no overlap of the maximum value in the frequency distribution plots with that of uninfected macrophages.

We have demonstrated that infected macrophages have significantly higher glycolytic fluxes and reduced PPP fluxes. The flux partitioning between glycolysis and PPP was altered upon infection, channelling higher carbon catabolism through glycolysis. Glycolytic glucose catabolism generates adenosine triphosphate (ATP) and nicotinamide adenine dinucleotide (NADH) which are important coenzymes/cofactors for biological functions and energy production in cells (Figure 6). The increase in glycolytic fluxes resulting in higher production of ATP and NADH might be required to fuel the immune responses against Mtb. Targeting the reprogrammed fluxes in infection could help improve TB treatment. For instance, if we could boost glycolytic fluxes in infected cells through therapies this could enhance the elimination of Mtb.

### Macrophages exhibited relatively low carbon fluxes through the TCA cycle

Fluxes through the TCA cycle were significantly lower compared to glycolysis. The TCA cycle generates co-enzymes that participate in the mitochondrial electron transport chain for ATP production. Macrophages are non-diving cells, so the relatively low TCA cycle fluxes indicate a low demand for carbon oxidation through this cycle and subsequent ATP production. Instead, the cellular energy and biosynthetic demand is met by glycolysis and the PPP fluxes. Thus, the TCA cycle fluxes are not the primary route for carbon oxidation in macrophages. Flux profiles show complete use of TCA cycle fluxes R21, R22, R24, R25, R26, R27, R28 and R29 in the oxidative phase (Figure 5). The frequency distribution plots in Supplementary Figure S3 shows no statistically significant differences in the TCA cycle and anaplerotic (malic enzyme and PEP carboxykinase) fluxes between uninfected and infected macrophages although the net fluxes were over 10 times higher in infected macrophages. These fluxes were statistically less well-resolved as compared to glycolysis and the PPP fluxes probably because of the choice of the ^13^C-substrate. Here we chose glucose as the substrate, which is primarily utilised through glycolysis and the PPP, and therefore, these fluxes are statistically well-resolved.

### Amino acid fluxes were reprogrammed in infected macrophages

**Figure 8:**
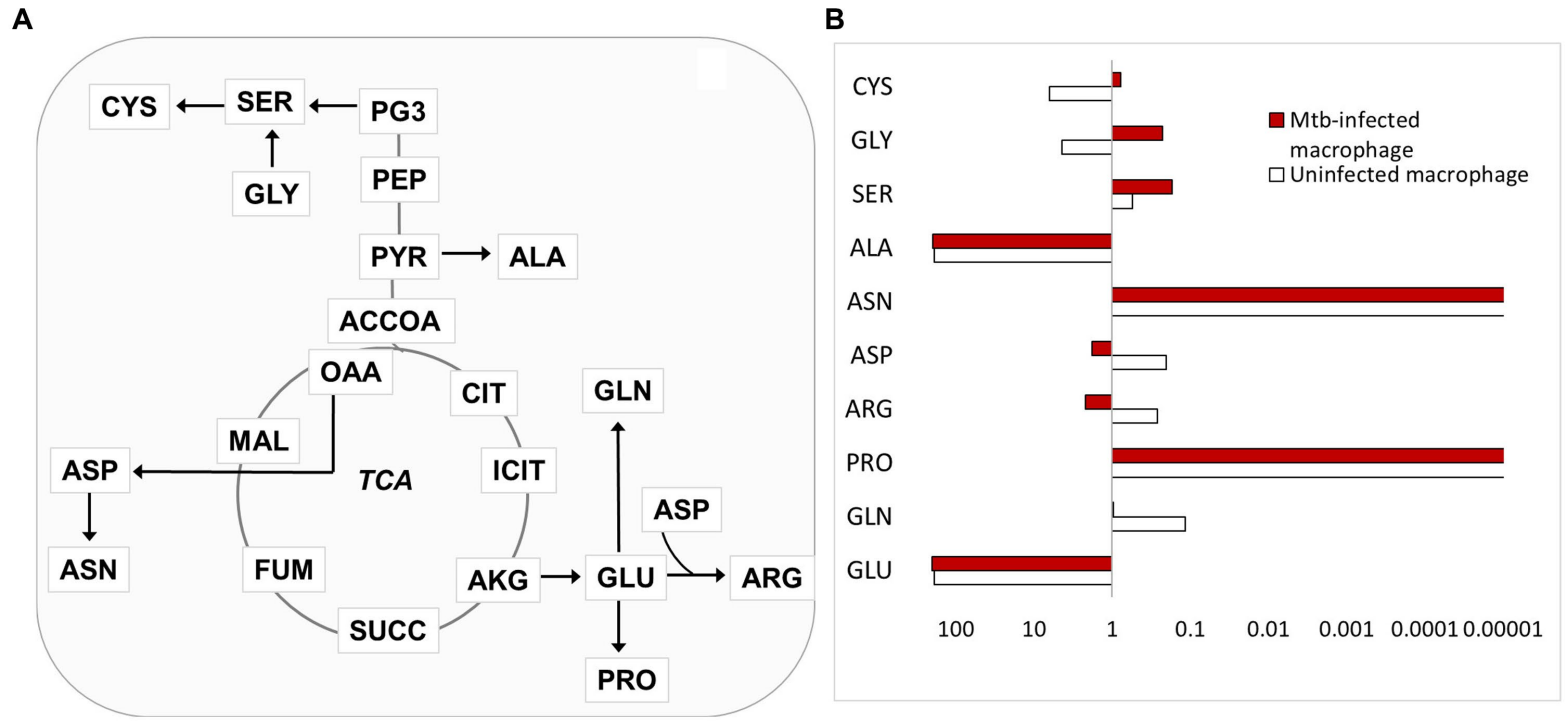
Carbon flux distributions in amino acid synthesis. **(A)** Outline of amino acid synthesis in macrophages. Serine (SER) is synthesised from PG3; cysteine (CYS) is synthesised from SER; glycine (GLY) is synthesised from SER, the arrow from GLY to SER indicates SER synthesis from GLY; alanine (ALA) is synthesised from PYR; glutamate (GLU) is synthesised from AKG; glutamine (GLN), proline (PRO) is synthesised from GLU; arginine (ARG) is synthesised from ASP and GLU. **(B)** Net fluxes relative to glucose uptake are shown for the amino acid metabolic fluxes represented in **A**.

We also quantified the carbon fluxes for non-essential amino acid biosynthesis (Figure 8). The TCA cycle and glycolysis are the primary sources of metabolic precursors required for the biosynthesis of amino acids. The TCA cycle-derived aspartate, arginine, glutamate, and glutamine and PYR-derived alanine biosynthetic fluxes were higher in infected macrophages. Aspartate, glutamate, and glutamine are acquired by the pathogenic Mtb as carbon and nitrogen nutrient sources during intracellular growth (Beste et al., 2013; Borah et al., 2019a). The precise role of these amino acids in macrophage metabolism and immunity still needs further investigation. The increased flux to arginine synthesis can be explained by the involvement of this amino acid in macrophage metabolism and immune responses against Mtb (McKell et al., 2021). Proline and asparagine fluxes did not change in infected macrophages and tyrosine fluxes (not shown in Figure 8) were higher in infected macrophages. The fluxes to glycolytic intermediate PG3-derived serine, cysteine, and glycine synthesis were reduced in infected macrophages (Figure 8). We have previously demonstrated that serine from host macrophages is not available to intracellular Mtb (Borah et al., 2019a). Mtb with deletion in *de novo* serine biosynthetic pathway failed to survive in human THP-1 macrophages (Borah et al., 2019a). The reduced serine biosynthetic flux in host macrophages is a metabolic phenotype associated with proinflammatory M1 macrophages that restricts bacterial growth.

## Discussion

Host-pathogen metabolic interactions are important in TB. The TB pathogen adapts its metabolism and acquires nutrients during growth in human macrophages (Beste et al., 2013; Borah et al., 2019a,b; Chandra et al., 2020). Previous work has reported that Mtb infection induces metabolic reprogramming in infected macrophages (Gleeson et al., 2016; Cumming et al., 2018). The metabolic reprogramming is intimately linked to the polarisation of macrophages to M1 and M2 functional phenotypes (Shi et al., 2016). Metabolic fluxes are core to facilitating this reprogramming, and the knowledge of the infected host’s metabolic fluxes could ultimately provide new targets for therapeutic development. In this study, we applied ^13^C-MFA to measure the intracellular carbon metabolic flux reprogramming of Mtb-infected human THP-1 macrophages.

We used human THP-1 macrophages as the *in vitro* infected host model for conducting ^13^C-MFA. THP-1 monocytic cell line is a frequently used model in TB research and other areas. Other studies have investigated the relevance of THP-1 as a human macrophage model (Qin, 2012; Tedesco et al., 2018). The THP-1 macrophages showed similar mitochondrial respiratory and glycolytic extracellular acidification profiles as human monocyte-derived macrophages when infected with pathogenic Mtb H37Rv (Cumming et al., 2018). As our study is the first application of ^13^C-MFA in human macrophages, we chose THP-1 as the macrophage model to minimise heterogeneity and variations between cell populations which exist with primary human monocyte-derived macrophages isolated from different individuals (Qin, 2012; Tedesco et al., 2018).

We quantified intracellular carbon metabolic fluxes of uninfected and Mtb-infected macrophages. Mtb infection leads to an increase in glycolytic fluxes, which is the characteristic metabolic phenotype of M1 proinflammatory microbicidal macrophages (Gleeson et al., 2016). Glycolytic fluxes are important to mount appropriate immune responses to control Mtb’s growth in macrophages (Gleeson et al., 2016). Overexpression of glucose transporter-1 (GLUT1) has been demonstrated to increase glycolysis and proinflammatory cytokine interleukin (IL)-6) and tumour necrosis factor (TNF)-α production (Gauthier and Chen, 2022). The shift of metabolic phenotype from oxidative phosphorylation (OXPHOS) to aerobic glycolysis in M1 macrophages is mediated by hypoxia inducible factor (HIF-1α) (Wang et al., 2017; Marín Franco et al., 2020; Terán et al., 2022). In extrapulmonary pleural TB, inhibition of HIF-1α and changes in macrophage phenotype from glycolysis to OXPHOS resulted in attenuation of microbicidal properties of infected macrophages and the consequent failure to restrict infection. Restoration of HIF-1α activity increased glycolysis in macrophages and in C57BL/6 mice resulting in better control of Mtb (Marín Franco et al., 2020). In addition to macrophages other immune cells, such as tissue-resident innate lymphoid cells and CD8+ T cells showed an increased metabolic dependency on glycolysis upon Mtb infection, which highlights the intimate link between glycolysis and immune responses across multiple immune cells (Russell et al., 2019; Corral et al., 2022).

The increased flux through glycolysis is directly linked to the increase in the total cellular carbohydrates of infected macrophages. The glycolytic flux profile of THP-1 macrophage is relevant to the metabolic changes measured in other *in vitro* (primary macrophages) and *in vivo* models of TB using other omic tools. Metabolite profiling using ^1^H-NMR (nuclear magnetic resonance) spectroscopy in C57Bl/6 mice infected with pathogenic Mtb showed elevated levels of glucose and lactate indicating higher glycolytic fluxes upon Mtb infection (Shin et al., 2011). A different study demonstrated glycolytic differences in primary macrophages infected with virulent and non-virulent Mtb (Cumming et al., 2018). This study showed a reduced glycolytic extracellular acidification rate in Mtb-infected macrophages which contrasts with ours and other studies that demonstrate significant glycolytic dependency of the host when tested in *in vitro* macrophages and *in vivo* mice models. The differences in the observations could be attributed to the different experimental set up used. This study used an infection period of 18 h followed by 8 h of ^13^C-glucose labelling, which is probably not enough to reach steady state labelling in these cells. Our ^13^C-glucose labelling of Mtb-infected macrophages was conducted for 48 h which was established as the pseudo steady-state period where the cells have reached isotopic steady state labelling; there were no significant changes in ^13^C-labelling after 48 h which indicate that the macrophages have achieved metabolic steady state (Borah et al., 2019a).

The glycolytic fluxes lead to pyruvate synthesis which is primarily utilised for lactate and/or alanine synthesis. We found that the total flux from pyruvate to lactate/alanine production was significantly higher in Mtb-infected macrophages. Others have also shown higher levels of lactate production in human alveolar and monocyte-derived macrophages, and murine bone marrow derived macrophages when infected with Mtb (Gleeson et al., 2016). Mtb uses host cell lactate during infection as a carbon substrate; this was demonstrated by the attenuation of an Mtb strain unable to oxidise lactate in primary macrophages (Billig et al., 2017). Thus, lactate is one of the key metabolites for host-pathogen interaction in TB but is a debatable target for HDT. Lactate can be used to refuel glycolysis and its synthesis is important to control Mtb’s growth, so inhibiting lactate production in the host is not a potential target (Kiran and Basaraba, 2021). However, compounds that could target Mtb’s lactate synthesis specifically could be a potential alternative to develop anti-TB therapies.

The higher fluxes through glycolysis were accompanied by reduced PPP fluxes in infected macrophages. The PPP produces NADPH and ribose 5-phosphate biosynthetic precursors required for cell proliferation, nucleotide metabolism and lipid synthesis. The reduced carbon fluxes through the PPP suggest the redistribution of a large proportion of carbon flux through glycolysis to generate energy for fuelling immune responses against Mtb whilst reducing the carbon flux to biomass production. The mitochondrial TCA cycle is a critical pathway to generate substrates and precursors for ATP production in a cell. M1 macrophages are characterised by a low dependency on the TCA cycle and OXPHOS. The mitochondrial energy metabolism was significantly reduced in macrophages and T-cells infected with Mtb (Cumming et al., 2018; Russell et al., 2019). Our flux maps show relatively low carbon fluxes through the TCA cycle in uninfected and infected macrophages. Although statistically insignificant, the net flux values for the TCA cycle in infected macrophages were higher, suggesting subsequent activity of the mitochondrial OXPHOS. A recent study using human macrophage and zebra fish model of TB established that Mtb infection increased mitochondrial OXPHOS which was required to prevent mitochondrial damage at early stages of infection (Pagán et al., 2022). Here we show that the TCA cycle fluxes are active in infected macrophages although it is not the primary carbon utilising pathway.

Macrophage amino acid fluxes were reprogrammed upon Mtb infection. Serine, glycine, and cysteine fluxes were reduced in infected cells; these three amino acids share a common biosynthetic pathway. Our previous work established that serine is not available to intracellular Mtb; a serine auxotroph of Mtb failed to survive in macrophages (Borah et al., 2019a). The fact that fluxes to serine synthesis was reduced in infected macrophages could be a strategy to limit nutrition to Mtb and restrict its growth. Serine is an important amino acid in cell metabolism; this amino acid is involved in one-carbon, glutathione, nucleotide, and coenzyme (NADH and ATP) metabolism (Gauthier and Chen, 2022). The role of this amino acid in TB needs to be further investigated as it can lead to the development of potential HDTs.

Mtb acquires aspartate, glutamine, and glutamate from host macrophages; these amino acids are both carbon and nitrogen sources for intracellular Mtb (Beste et al., 2013; Borah et al., 2019a). The fluxes for aspartate, glutamine and glutamate synthesis are elevated in infected macrophages. Glutamine was demonstrated to be important for M1 macrophage polarisation; inhibition of glutamine catabolism reduced macrophage polarisation when tested in Mtb-infected murine bone marrow-derived macrophages (Jiang et al., 2022). Aspartate and glutamate are central precursors for the synthesis of other amino acids, and they participate in central metabolism. However, the roles of these two amino acids in macrophage immunometabolism are unclear and need further assessment. Arginine derived from aspartate and glutamate had elevated biosynthetic flux in infected macrophages. Arginine catabolism leads to the generation of nitric oxide which controls Mtb’s growth and reduces tissue damage in TB granulomas (Duque-Correa et al., 2014). The flux for alanine synthesis was also higher in infected macrophages. Alanine of host macrophages is used by Mtb and incorporated directly into its biomass (Borah et al., 2019a). The levels of these amino acids were found to be higher in the lungs of Mtb-infected mice compared to the uninfected (Shin et al., 2011). The increased levels of free amino acids have been linked to the impairment of protein synthesis, i.e., amino acids are used for metabolic processes rather than protein anabolism (Shin et al., 2011). We found that the total cellular protein content was lower in infected macrophages; this is probably associated with the higher amino acid metabolic fluxes that are involved in driving immunometabolism and immune functions, but the precise link still needs to be understood.

## Conclusion

In this study we present metabolic flux reprogramming of Mtb-infected human THP-1 macrophages. We conducted ^13^C-MFA to derive carbon flux profiles that drive cellular metabolism and function. Glycolytic fluxes were significantly higher leading to increased pyruvate metabolism, and an overall increase in total cellular carbohydrate. The PPP fluxes were reduced upon infection. The TCA cycle-derived amino acid fluxes were higher and serine synthesis was reduced in infected macrophages. The Mtb-infected macrophage flux profile highlights the key nodes that are reprogrammed during infection and are the targets for further investigation. This may lead to the development of new therapies such as adjuvants to be used with antimicrobials to improve infection outcomes in TB patients.

## Data availability statement

The original contributions presented in the study are included in the article/Supplementary material, further inquiries can be directed to the corresponding author.

## Ethics statement

Ethical approval was not required for the studies on humans in accordance with the local legislation and institutional requirements because only commercially available established cell lines were used. Ethical approval was not required for the studies on animals in accordance with the local legislation and institutional requirements because only commercially available established cell lines were used.

## Author contributions

KB: Conceptualization, Formal analysis, Funding acquisition, Investigation, Methodology, Project administration, Resources, Supervision, Visualization, Writing – original draft, Writing – review & editing. LM: Formal analysis, Writing – review & editing. YX: Formal analysis, Writing – review & editing. DK: Writing – review & editing.
